# How to have a metalinguistic dispute

**DOI:** 10.1007/s11229-021-03038-2

**Published:** 2021-03-15

**Authors:** Poppy Mankowitz

**Affiliations:** Department of Philosophy, Cotham House, University of Bristol, Bristol, BS6 6JL United Kingdom

**Keywords:** Verbal disputes, Metalinguistic disputes, Metalinguistic negotiation, Focus, Philosophy of language, Pragmatics

## Abstract

There has been recent interest in the idea that speakers who appear to be having a verbal dispute may in fact be engaged in a *metalinguistic negotiation*: they are communicating information about how they believe an expression should be used. For example, individuals involved in a dispute about whether a racehorse is an athlete might be communicating their diverging views about how ‘athlete’ should be used. While many have argued that metalinguistic negotiation is a pervasive feature of philosophical and everyday discourse, the literature currently lacks an account of this phenomenon that can be situated within a ‘mainstream’ view of communication. I propose an independently motivated account where individuals reconstruct metalinguistic propositions by means of a pragmatic, Gricean reasoning process.

## Introduction

Disputes sometimes seem *verbal*, which roughly means that the participants ‘agree on the relevant facts about a domain of concern and just disagree about the language used to describe that domain’ (Chalmers 2011, p. 515).^1^ The following is a candidate for a verbal dispute, where A (a speaker of American English) and B (a speaker of British English) agree that the bowl contains what they would respectively describe as ‘chips’ and ‘crisps’: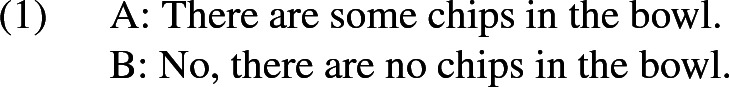 It is natural to conclude from these sorts of examples that verbal disputes are non-substantive, insofar as the participants disagree over some trivial matter and are simply talking past each other. In light of arguments that verbal disputes are pervasive within philosophical debates (see Carnap 1950; Putnam 1987; Hirsch 2005, 2011; Chalmers 2011), the view that verbal disputes are non-substantive could have significant ramifications for philosophy.

Yet it has recently been argued that verbal disputes may be substantive at the metalinguistic level (Plunkett and Sundell 2013, 2014, 2019; Plunkett 2015; Sundell 2016). Plunkett and Sundell (2013, p. 3) begin by defining a *metalinguistic usage* of a linguistic expression, which arises when it is ‘used (*not* mentioned) to communicate information about the appropriate usage of that very expression in context’.^2^ They call a dispute that centres on the propriety of expressions employed in a metalinguistic usage a ‘*metalinguistic dispute*’. A *descriptive* metalinguistic dispute concerns how an expression *is* used in the relevant context, whereas a *normative* metalinguistic dispute concerns how an expression *should be* used in the relevant context, a matter that may be independent of how the word is actually used. They coin the phrase ‘*metalinguistic negotiations*’ to refer to normative metalinguistic disputes. A metalinguistic negotiation would plausibly count as substantive, since the disputants are engaged in an activity more sophisticated than merely talking past each other, and there may be non-trivial, real-world implications of different choices about how to use language.

Plunkett and Sundell provide the following examples of metalinguistic negotiations:
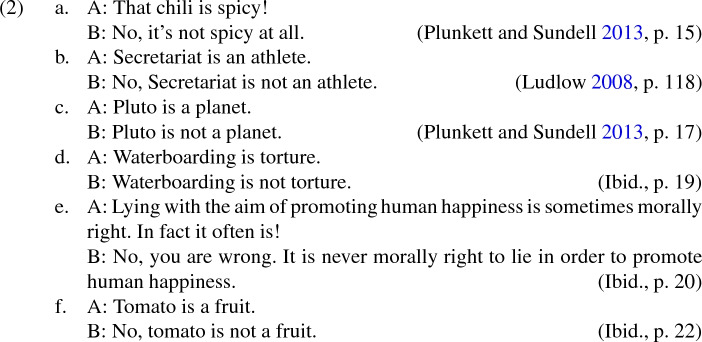
 Plunkett and Sundell (2013, pp. 15–16) take (2a) and (2b) to be metalinguistic negotiations because participants in the dispute ‘agree on what the chili actually tastes like’ and ‘mutually know all of the facts about [the racehorse] Secretariat’s speed, strength, etc., and what races, awards, medals he won, etc.’, but disagree about how the words ‘spicy’ and ‘athlete’ should be used. They endorse parallel analyses for (2c)–(2f).

Those who have discussed metalinguistic negotiation consider it to be a pervasive feature of philosophical and ordinary discourse, including discussions about aesthetics (Sundell 2011), metaphysics (Thomasson 2015, 2017; Belleri 2017), law (Plunkett and Sundell 2014), ethics (Plunkett and Sundell 2013; Plunkett 2015; Rast 2017), and other everyday topics. Yet the literature lacks a detailed analysis of metalinguistic negotiation situated within a ‘mainstream’ theory of communication. In the current paper, I provide such an analysis for those who wish to classify certain disputes as metalinguistic negotiations; though I remain neutral about whether any particular dispute is plausibly analysed as a metalinguistic negotiation.^3^

The proposed analysis holds that assessors use a Gricean reasoning process to reconstruct a metalinguistic proposition, where this metalinguistic proposition states that certain expressions are apt for using as part of conveying a contextually determined proposition. This analysis applies to both descriptive and normative metalinguistic disputes: an expression might be apt for conveying a proposition due to factors related to how expressions *are* used by speakers, or due to factors related to how expressions *should be* used. Moreover, the analysis is based on an independently motivated account of the phenomenon of expression focus, which I take to be methodologically preferable to the development of an account specifically designed to handle metalinguistic disputes.

In Sect. 1, I provide an overview of the existing literature on metalinguistic negotiation and outline three conditions for an adequate account. In Sect. 2, I explain the appeal of two central features of the account to be developed, and in Sect. 3, I present the account.

## Existing accounts

An adequate account of metalinguistic negotiation must be given if the notion is to be used to explain how verbal disputes can be substantive. It is reasonable to expect an adequate account to achieve the following:
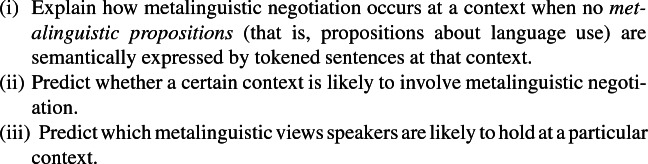
 For example, if (2a) involves metalinguistic negotiation, then an adequate account should, firstly, explain how this occurred, given that neither speaker tokened sentences that semantically express propositions about how language should be used. Secondly, it should predict that metalinguistic negotiation is likely to occur at the context evoked by (2a). Thirdly, it should predict that speaker A is likely to think that ‘athlete’ *should* be used with respect to Secretariat at the relevant context, whereas B is likely to think that ‘athlete’ *should not* be used with respect to Secretariat. Note that the formulation of (iii) is neutral on the question of whether participants in metalinguistic negotiations convey their metalinguistic views by communicating metalinguistic propositions; I will describe accounts that affirm this as ‘*propositional*’, and accounts that deny it as ‘*non-propositional*’.

Since Plunkett and Sundell (2013, fn. 11) ‘aim to demonstrate that the metalinguistic analysis [...] is consistent with, and indeed highly plausible on, entirely mainstream views of linguistic communication’, I am exclusively interested in adequate accounts that are situated within a ‘mainstream’ linguistic theory, by which I mean a theory that takes a significant proportion of ordinary conversations to be devoid of metalinguistic negotiation.^4^ To my knowledge, the only attempts in the literature to situate an account within a ‘mainstream’ theory of communication occur in Belleri 2017 and Thomasson 2017. I now argue that neither Belleri’s propositional account nor Thomasson’s non-propositional one meets the adequacy conditions.

Belleri (2017, p. 2215) holds that ‘the pragmatic inference that would allow each participant to go from the verbal, object-level disagreement to the implicitly communicated metalinguistic disagreement [...] could involve charity as well as broadly construed Gricean considerations as to what best explains the assumed cooperativeness of the speaker’. She further clarifies that her preferred account treats metalinguistic negotiations as ‘*conversationally implicating* normative claims as to which linguistic options should be favoured’ (fn. 10). Grice and the neo-Griceans (e.g., Atlas and Levinson 1981; Horn 1984; Levinson 2000) hold that cooperative discourse is governed by maxims, which speakers may deliberately violate in order to convey information distinct from what is semantically expressed by their utterances. When a speaker appears to violate a maxim in uttering a sentence, but the hypothesis that the speaker is obeying the maxims may be upheld by supposing that she thinks (and expects hearers to be able to work out that she thinks) that *p*, then the speaker has *conversationally implicated* *p*. Belleri sketches the sort of Gricean reasoning process that might occur during a verbal dispute on the topic of the persistence of objects through time: the hearer takes the fact that the speaker has issued a claim that is obviously false according to the hearer’s views to indicate that the speaker is using terms with different meanings; given that the speaker should know that she is using terms with different meanings, the hearer infers that the speaker must be conveying that the hearer should adopt the speaker’s way of using terms.

I will end up agreeing with Belleri’s proposal that metalinguistic negotiations involve conversationally implicating metalinguistic propositions. However, while Belleri sketches how this procedure might work for one particular case, she does not provide general principles that explain how individuals access metalinguistic propositions for all cases. Moreover, she neither predicts when individuals will be able to access metalinguistic propositions, nor exactly which metalinguistic propositions will be accessed in such cases.

Thomasson’s non-propositional approach takes metalinguistic negotiations to arise when ‘speakers use terms in certain ways in order to reinforce or alter the semantic norms of their use’, rather than ‘to communicate the speaker’s belief about how the term should be used (though the hearer may nonetheless legitimately infer those beliefs)’ (2017, p. 25). She claims that a major point in favour of this approach is its potential to explain why speakers would engage in metalinguistic negotiations in the object language, rather than issuing explicit metalinguistic claims: ‘if the goal is to modify the norms governing the use of the term, the best way to do that may be *to present themselves as making a discovery about the world* [...] rather than presenting themselves as making their own recommendations about how to change how we use terminology’ (Ibid.).

As Thomasson acknowledges, she does not provide a detailed explanation of the means by which metalinguistic negotiations modify norms. Hence predictions do not emerge about when metalinguistic negotiations are likely to occur, or which metalinguistic beliefs hearers will be likely to infer. Moreover, it is not only advocates of her view who are able to explain why speakers would engage in metalinguistic negotiations in the object language. A similar explanation, compatible with propositional accounts, may be found in Plunkett and Sundell 2014: ordinary speakers tend to have ‘a suspicion of disagreements about language [...] [and] will thus be resistant to any analysis that seemingly reduces the debate to a dispute about language’ (p. 70). By refraining from explicitly stating the metalinguistic propositions that they implicate, speakers may therefore maintain that they are engaged in a debate about something other than language.

Plunkett and Sundell themselves ‘have been content to remain neutral’ (2019, p. 12) on the mechanisms that underlie metalinguistic negotiation. They do not even take a position on whether propositional or non-propositional accounts are preferable. As observed by Thomasson (2017, pp. 22–23), ‘[a]t certain points, they speak in ways consistent with the conversational implicature reading of metalinguistic negotiation, speaking of speakers as ‘communicating’ ‘information’ via metalinguistic usage [...] [M]ore commonly they refer to speakers as ‘advocating’ for certain ways of using a term’. Plunkett and Sundell (2019, p. 12) briefly sketch (without endorsing) a Gricean analysis of metalinguistic negotiation, claiming that the hearer would ‘reason—in a manner similar to how Grice describes a quality implicature—that the speaker would only have said the thing they did, using the terms in the way they did, if they (the speaker) believed that using the terms in that way was appropriate under the circumstances’. Yet they raise doubts about the potential to situate this sketch within a standard Gricean framework, since ‘[e]xpressing a proposition [...] that carves up the world in some inappropriate way or uses words in a way that we know to have pernicious effects is clearly uncooperative, but it doesn’t violate [the maxims of Quality]’ (Plunkett and Sundell 2019, fn. 13). This indicates that developing an analysis of metalinguistic negotiation within a Gricean approach is a non-trivial feat. The analysis that I develop diverges from those sketched by Belleri and Plunkett and Sundell, since it emphasises the role of other maxims in addition to those of Quality, while setting out a precise description of the hearer’s reasoning process.

In sum, there have been few attempts at providing an analysis of metalinguistic negotiation within a ‘mainstream’ linguistic theory, and those accounts that have been suggested fail to meet three reasonable adequacy conditions.

## Preliminaries for an account

The account of metalinguistic negotiation to be developed in Sect.  3 has two features that are desirable for any adequate account. First, it is a propositional account, and second, it is based on an independently motivated account of expression focus. I explain each of these features in turn.

### Propositional accounts

The literature lacks a comparison of the merits of accounts’ affirming and denying that metalinguistic propositions are conveyed during metalinguistic disputes. I want to draw attention to a compelling reason in favour of propositional accounts. Observe how some metalinguistic negotiations involve the tokening of sentences that contain *sentential operators* (expressions that apply to a sentence to produce another expression such as a sentence; e.g., ‘sometimes’, ‘Yasma thinks that’, etc.):
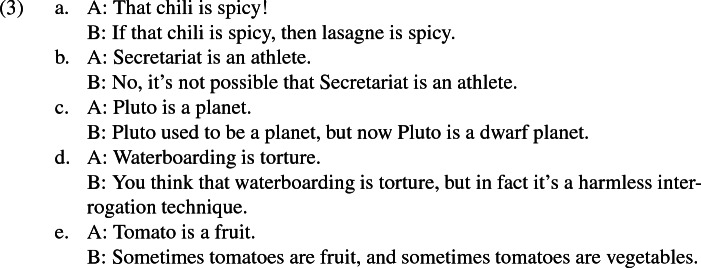
These disputes are naturally understood to possess the features that Plunkett and Sundell take to characterise metalinguistic negotiations: the relevant expression (‘athlete’, etc.) is used to communicate information about the appropriate usage of that very expression, and the disputes concern how the expression should be used relative to certain contexts. In canonical examples of metalinguistic negotiations like (2a)–(2e), speakers’ views were simple affirmations or denials of the propriety of using an expression in a certain way at their context. Yet in (3a)–(3e), B is naturally understood to hold more complex views about the propriety of using the relevant expression. Moreover, sentential operators that occur in B’s utterances determine exactly which complex view B appears to hold. For example, in (3a) B is naturally understood to hold the view that, *if we should* use ‘spicy’ with respect to the chili for certain purposes, *then we should* use ‘spicy’ with respect to lasagne for certain purposes.^5^ In (3e), B is naturally understood to think that *sometimes we should* use ‘fruit’ with respect to tomatoes for certain purposes, and *sometimes we should* use ‘vegetables’ with respect to tomatoes for certain purposes.

It is unclear how the complex views that B appears to hold in (3a)–(3e) could be captured in non-propositional terms, such as through B’s altering semantic norms. In contrast, propositional accounts can easily handle these cases: while participants in the canonical examples of metalinguistic negotiations pragmatically convey propositions that affirm or deny the propriety of using the relevant expressions in certain ways, these propositions can interact with the meanings of tokened sentential operators in order to form propositions consisting of more complex claims about appropriate language use.

Against this reasoning, it might be claimed that pragmatically conveyed content cannot interact with tokened sentential operators. It would follow that, if (3a)–(3e) convey complex metalinguistic propositions to which the meanings of sentential operators contribute, then they must semantically express these propositions.^6^ There are several good reasons to resist this position. First, it is intuitively implausible to claim that (say) ‘Sometimes tomatoes are fruit’ *means* that sometimes we should use the expression ‘fruit’ with respect to tomatoes for certain purposes; it seems to semantically express something about tomatoes, rather than about appropriate language use. Second, the view would entail either that any sentence that can be used in a metalinguistic dispute (e.g., ‘Tomatoes are fruit’) also semantically expresses a proposition about language use, or that adding a sentential operator to a sentence that expresses a non-metalinguistic proposition is sufficient to cause it to express a metalinguistic proposition, where both of these options seem unnatural. Third, Plunkett and Sundell (2019, p. 5) are clear that during metalinguistic disputes, ‘the content about which [the speakers] disagree isn’t the semantic content of their expressions in their context’; hence if metalinguistic propositions are semantically expressed in (3a)–(3e), then we would be unable to classify them as metalinguistic negotiations, despite the fact that they have the characteristics identified by Plunkett and Sundell. (3a)–(3e) thus justify the inference that pragmatically conveyed content *can* interact with tokened sentential operators, especially if a plausible account of this interaction is given (see Sect. 3.3).

In sum, anyone who concedes the possibility of metalinguistic negotiations like (3a)–(3e) has a compelling reason to accept a propositional account. Accordingly, the account that I go on to develop will be propositional.

### Independent motivations and expression focus

It is desirable for an account of metalinguistic negotiation to be based on an independently motivated account of some broader or related phenomenon, if possible. This not only accords with the aim of theoretical parsimony, but also means that the motivation to accept the proposed account is partially independent of considerations related specifically to metalinguistic negotiations. I will suggest that there are connections between metalinguistic negotiation and the phenomenon of expression focus. After giving an overview of focus and explaining its connection to metalinguistic usage, I will sketch the central features of an account of expression focus and show how they extend to metalinguistic negotiation.

The *focus* of an occurrence of a sentence is an expression that is marked with vocal emphasis in spoken form, and which indicates that certain alternatives to that expression are relevant to our understanding of the sentence.^7^ Ordinarily, focus is used to draw attention to alternatives to the denotation of the focused item. For example, where ‘[ $]_F$’ marks the constituent in focus and capitalised morphemes are to be read with vocal emphasis, alternative properties that Grandpa may have are relevant to our understanding of (4a), whereas alternative individuals that may have died are relevant to our understanding of (4b):
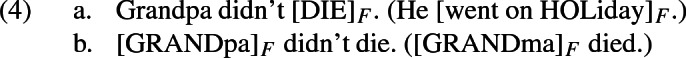
*Expression focus* (see Wedgwood 2005; Krifka 2007; Li 2017) is a special type of focus that speakers utilise to draw attention to alternative linguistic items rather than denotations. For instance, alternative expressions that may be used as part of conveying that Grandpa died are relevant to our understanding of (5a). In (5b) and (5c), the relevant alternative expressions are ones that may be used as part of conveying that individuals eat rutabaga and that some geese are flying:
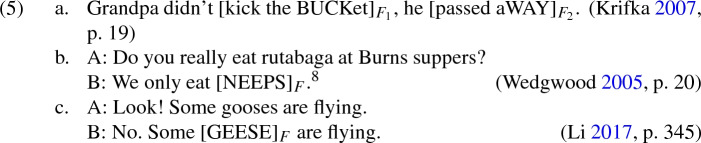
8

There are two main reasons to postulate a connection between expression focus and metalinguistic disputes. Firstly, whenever an expression involves expression focus, it will meet Plunkett and Sundell’s criteria to count as a metalinguistic usage. For example, in (5a) ‘kick the bucket’ is used to communicate information about the appropriate usage of that expression, specifically that the speaker thinks that the expression is inappropriate to use as part of conveying that Grandpa died. Secondly, when a metalinguistic usage of an expression occurs, it is natural for that expression to be focused. For instance, Plunkett and Sundell’s intended construal of (2b) is most naturally attained via vocal emphasis on the occurrences of ‘athlete’.^9^ On the other hand, the following vocal emphasis suggests that the speakers are concerned with establishing some examples of athletes, rather than how the expression ‘athlete’ should be used:
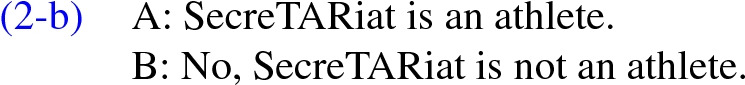
These observations indicate a close connection between expression focus and metalinguistic usage of expressions. Yet it need not follow that an expression is employed in a metalinguistic usage *if and only if* it receives expression focus. A reasonable position is that certain contextual features might allow assessors to infer that an occurrence of an unfocused expression is employed in a metalinguistic usage, but this is less natural and straightforward than the inference that an occurrence of a focused expression is employed in a metalinguistic usage.

Accordingly, I adapt the account of expression focus developed in Mankowitz 2020, which describes how speakers can convey metalinguistic propositions about the aptness of focused expressions in certain contexts. Informally, the account of expression focus takes these metalinguistic propositions to concern the aptness of using the focused expression as part of conveying a non-metalinguistic, *embedded proposition* (so-called to distinguish it from the metalinguistic proposition to which it contributes). More formally, the account defines an *aptness relation*, which holds between an expression *e* and a proposition *p* at a circumstance of evaluation *i* if and only if *e* is apt for using as part of conveying *p* at *i*, relative to contextually relevant standards.^10^ A metalinguistic proposition stating that an expression *e* is apt for conveying an embedded proposition $\alpha (\beta )$ is then formulated as: Apt$(e)(\alpha (\beta ))$.

Two questions about embedded propositions remain to be addressed. First, one might wonder why metalinguistic propositions concerning the aptness of expressions should be thought to include embedded propositions in the first place. The answer is that the use of an expression can reasonably be considered appropriate or inappropriate at a circumstance only relative to the conveying of particular information. While Plunkett and Sundell often imply that speakers hold absolute views about the propriety of using an expression with respect to some non-linguistic item, this cannot be an accurate depiction of speakers’ views. For example, Plunkett and Sundell (2013, p. 15) describe (2a) as a case where A ‘accepts the content that *we should use ‘spicy’ in such a way that it applies to the chili*’. Yet speaker A would presumably consider it *inappropriate* to use ‘spicy’ in order to convey that the chili is mild, brown, or a tedious topic of conversation. Speaker A’s view that it is appropriate to use ‘spicy’ with respect to the chili is really the view that it is appropriate to use ‘spicy’ *as part of conveying that the chili has certain properties* (more specifically, a particular flavour). It is therefore natural to accept that the propriety of an expression is always implicitly relativised to the conveying of some information. This idea is captured by taking metalinguistic propositions about appropriate language use to concern an aptness relation between an expression and a proposition.

Second, one might wonder how embedded propositions are formed. In order to capture the full range of metalinguistic propositions that can be conveyed by means of uses of expression focus, the account under discussion takes the context to supply a salient non-linguistic item that forms the embedded proposition when combined with the denotations of unfocused expressions. An item is *salient* when it is ‘the focus of perceptual or cognitive attention’ with respect to the discourse participants at a context (Mount 2008, p. 154). Generalising, a metalinguistic proposition Apt$(e)(\alpha (\beta ))$ conveyed by a participant in a metalinguistic dispute will be formed by combining a contextually salient non-linguistic item $\alpha$ with the denotation $\beta$ of expressions employed in non-metalinguistic usages.^11^ For example, the speakers in (2b) communicate that ‘athlete’ is, or fails to be, apt for using as part of conveying the proposition that Secretariat has a salient property, such as being a successful racehorse. The fact that the account does not require the denotation of an expression *e* employed in a metalinguistic usage to contribute to the embedded proposition is appealing in light of Plunkett and Sundell’s view that metalinguistic negotiations often occur at contexts for which ‘there is no antecedently settled matter of fact about [*e*’s] meaning’ (2014, p. 64) and *e* thus lacks a unique denotation.

In sum, it is desirable to analyse metalinguistic negotiation by means of an independently motivated account of some related phenomenon. The connections between the phenomena of metalinguistic negotiation and expression focus additionally render it desirable to analyse the former by means of an account of the latter. Adapting an existing account of expression focus allows the views communicated by participants in metalinguistic disputes to be analysed as metalinguistic propositions stating that an aptness relation holds between the expression employed in a metalinguistic usage and a context-dependent, embedded proposition.^12^ The embedded proposition is formed by combining the denotations of expressions employed in non-metalinguistic usages with a salient non-linguistic item.

## An account of metalinguistic disputes

The analysis of expression focus in Mankowitz (2020) begins by showing how to formulate metalinguistic propositions concerning the aptness of expressions, before providing an account of the pragmatic reasoning process that allows hearers to reconstruct particular metalinguistic propositions on the basis of a speaker’s utterance. Section 2.2 gives sufficient detail about the structure of metalinguistic propositions for current purposes. This section extends the pragmatic component of the prior account, in order to implement the following view: metalinguistic disputes, including metalinguistic negotiations, arise when (real or imaginary) speakers are understood to conversationally implicate propositions about the aptness of using certain expressions as part of conveying contextually determined propositions. I first describe the pragmatic reasoning process that I attribute to assessors, breaking it down into four steps for clarity of presentation.^13^ I then apply it to a couple of paradigm metalinguistic negotiations.

### Step (i)

In step (i), an assessor doubts that the (real or imaginary) speaker is proposing to add the non-metalinguistic proposition expressed by her utterance to the common ground. Several aspects of this step require explanation.

First, I follow Stalnaker (1978) by modelling communication in terms of a *common ground*, which consists of the propositions mutually accepted by interlocutors at a particular context for the purposes of their conversation. A speaker’s utterance can be seen as a proposal to add the information she intends to communicate to the common ground. Next, I assume that, even when an assessor encounters an out-of-context sentence (e.g., an example written in a philosophy paper), she will assess it relative to an imaginary context.^14^ I also assume that an assessor will imagine a context that at least includes a speaker bound by the usual norms of cooperative discourse, as well as a common ground that contains the sort of propositions that the assessor would expect to be mutually assumed in an ordinary context. If further details of the envisaged context are specified when an out-of-context sentence is provided (e.g., that the speakers agree about all of Secretariat’s sports-related properties), then an assessor can be expected to additionally imagine that these details hold.

I assume an assessor’s default hypothesis to be that a speaker intends the proposition expressed by her utterance to be added to the common ground. This default hypothesis is reassessed if a speaker with this intention would violate a Gricean conversational maxim (see discussion in Sect. 1, and Grice 1989, pp. 22–40). There are two types of context that allow this default hypothesis to be reassessed. First, some disputes that are inferred to be metalinguistic occur relative to contexts where the hearer is aware that the speaker’s utterance expresses a proposition *p*, and the intention to add *p* to the common ground would violate a Gricean maxim. The proposition *p* would be derived from the semantic values of expressions that have antecedently settled meanings, in combination with the speaker’s preferred meanings for any expressions that lack antecedently settled meanings. Most commonly in such contexts, the violation pertains to either the first maxim of Quantity (‘Make your contribution as informative as is required (for the current purposes of the exchange)’) or the first maxim of Quality (‘Do not say what you believe to be false’).

Second, there might be contexts where there are no antecedently fixed meanings for at least one of the tokened expressions, and the speaker’s preferred meanings for those expressions fail to establish any proposition that the hearer is able to identify. During these sorts of metalinguistic disputes, an individual will often reassess the default hypothesis because the speaker’s utterance expresses no identifiable proposition whatsoever. For such a context, a speaker who was proposing to add a proposition expressed by her utterance to the common ground would violate the first maxim of Quantity, because her contribution is entirely devoid of information that the hearer could be expected to access.^15^

In sum, step (i) is initiated by an assessor’s awareness that a speaker would violate a Gricean maxim if she were proposing to add some non-metalinguistic proposition expressed by her utterance to the common ground. This leads the assessor to doubt that the speaker did intend to convey such a proposition.

### Step (ii)

In step (ii), an assessor becomes aware that the violation of Gricean maxims need not be attributed to the speaker if the speaker is understood to be using her utterance to convey a metalinguistic proposition. This metalinguistic proposition will concern the aptness of certain expressions in the sentence for conveying a contextually determined proposition about the denotation of other expressions in the sentence.

The question emerges of how the assessor identifies which metalinguistic proposition the speaker might intend to convey. The first step is to infer the expressions about which an aptness claim is being communicated, or (in other words) the expressions that are being employed in a metalinguistic usage. In the majority of cases, these will be the expressions that the assessor independently understands to be the focus of the speaker’s utterance (see Sect. 2.2). The assessor will construe particular expressions as the focus most easily when those expressions are marked as such, either through vocal emphasis (in spoken form), or italicisation, capitalisation or scare quotes (in written form). Even when no choice of focus is indicated for a sentence, there is good evidence that reading the sentence aloud or silently causes an assessor to choose points of emphasis and a corresponding focus, which will generally be a clause-final constituent for English subject-verb-object sentences.^16^

A second aspect of reconstructing the metalinguistic proposition consists of establishing the embedded proposition that Sect. 2.2 argued was involved in aptness claims. A natural proposal is that participants in a metalinguistic dispute have a mutual interest in working out how to convey some embedded proposition, but they hold divergent views about which expressions are appropriate for using in order to convey it. It is important that the context supplies a salient non-linguistic item of a suitable type to form a proposition by combining with the denotation of expressions employed in a non-metalinguistic usage. However, speakers and hearers need not be in a position to identify an expression that denotes this salient non-linguistic item. All that is required is that participants in the discourse could converge upon some plausible candidates for the embedded propositions that they have a mutual interest in working out how to convey.^17^

To summarise, in step (ii) the assessor entertains the hypothesis that the speaker is uttering the sentence in order to convey a metalinguistic proposition concerning the aptness of certain expressions in the sentence (normally those that are construed as the focus) for conveying a contextually determined proposition about the denotation of certain other expressions in the sentences (normally those that are construed as unfocused).

### Step (iii)

Step (iii) occurs exclusively in cases where the speaker has tokened a sentence that includes at least one sentential operator. It consists of the assessor’s establishing which of multiple reconstructed metalinguistic propositions it would be most plausible for the speaker to be intending to convey.

The reason that this step is required is that, even after completing step (ii), multiple metalinguistic propositions will still be available when the sentence contains sentential operators. This is the case because the formal component of the account in Mankowitz (2020) formulates metalinguistic propositions by means of aptness relations that can interact with the meanings of sentential operators. For instance, the presence of the operator ‘not’ in an occurrence of a sentence allows the reconstruction of a metalinguistic proposition stating that the relevant expression *is apt* for conveying that the items *fail to have* the salient property (where Apt takes scope over the meaning of ‘not’, as for Apt$(e)(\lnot [\alpha (\beta )])$); and it additionally allows the reconstruction of one stating that the expression *fails to be apt* for conveying that the items *have* the salient property (where the meaning of ‘not’ takes scope over Apt, as for $\lnot$Apt$(e)(\alpha (\beta ))$).

The view that the meanings of sentential operators interact with aptness relations is attractive in light of the array of metalinguistic propositions that speakers can convey via sentences that contain operators. For while it is natural to understand the complex metalinguistic negotiations given in (3a)–(3e) to involve the communication of metalinguistic propositions where the meanings of the operators take scope over Apt (e.g., that *sometimes* the expression ‘fruit’ *is apt* for conveying a contextually determined proposition about tomatoes), other metalinguistic disputes are naturally understood to involve the communication of metalinguistic propositions where Apt takes scope over other operators.^18^ The fact that different contexts affect which metalinguistic proposition is reconstructed suggests that assessors use contextual clues to select the most plausible metalinguistic proposition when the speaker’s sentence includes sentential operators. Hence step (iii) consists of an assessor’s use of such contextual clues.

### Step (iv)

In the final step, the assessor concludes that the speaker implicates the metalinguistic proposition that the assessor has reconstructed during the preceding two steps. This conclusion is justified by the assessor’s awareness that the speaker knows that the assessor is capable of working through the preceding three steps, hence the speaker’s status as a cooperative discourse participant is compatible with his intending to communicate this metalinguistic proposition. If a speaker who intended to communicate this proposition would violate further conversational maxims, then the assessor must consider additional hypotheses about the metalinguistic or non-metalinguistic proposition that the speaker intended to convey, or abandon the assumption that the speaker is cooperative.

### Examples

I now describe a reasoning process that results in the metalinguistic construals of one of Plunkett and Sundell’s examples, (2b), along with one of my examples, (3c). In each case, I show how hearers may reason with respect to B’s utterance, although parallel reasoning could occur with respect to A’s utterance (with the omission of step (iii), when there are no sentential operators). While the described reasoning strikes me as a particularly natural way for an assessor to be led to access a metalinguistic proposition, there might be some occurrences of (2b) and (3c) for which assessors access a metalinguistic proposition via a reasoning process where another maxim risks being violated (e.g., the maxim of Relation, ‘Be relevant’).

First, my account holds that hearers or assessors who encounter (2b) may reason as follows, when the context supports the initiation of such a reasoning process (i.e., due to agreement about Secretariat’s sports-related properties):^19^
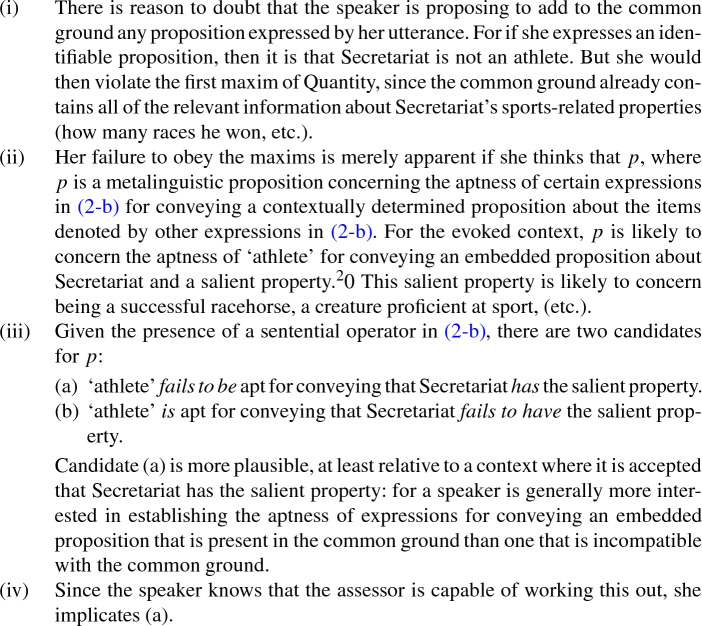
20 Next, my account holds that hearers or assessors who encounter (3c) may reason as follows, when the context supports the initiation of such a process:^21^
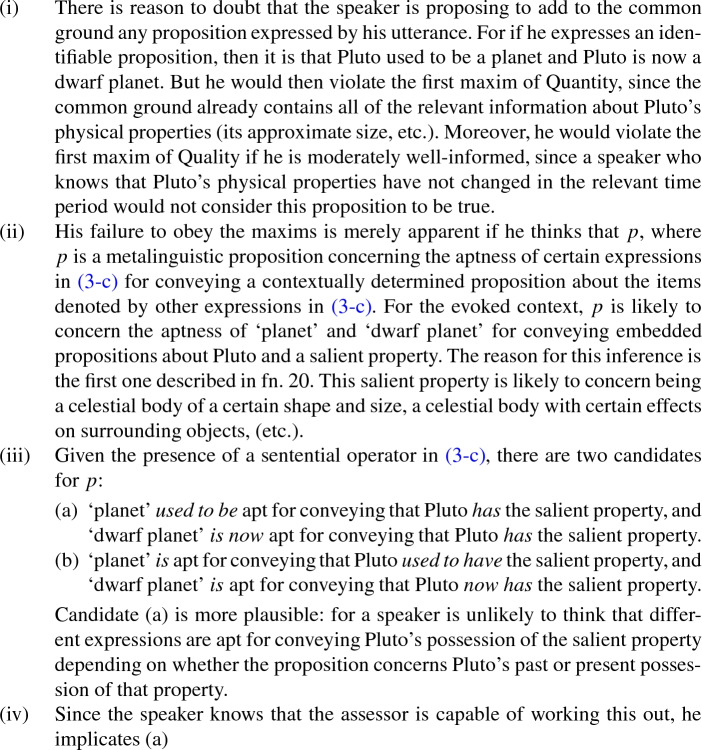


## Conclusion

Given the increasing interest in metalinguistic negotiation as a pervasive feature of discourse, it is surprising that the existing literature lacks a developed account situated within a ‘mainstream’ linguistic theory. I identified three adequacy conditions for an account, before explaining the appeal of developing a propositional account based on an independently motivated analysis of expression focus. I then described my account of metalinguistic disputes, including metalinguistic negotiations. While this account provides resources for those who wish to give a metalinguistic analysis of certain disputes, I take no position on whether any particular dispute should be analysed as a metalinguistic negotiation.

My account meets all three adequacy conditions. First, it explains how metalinguistic negotiation occurs at a context despite the fact that no metalinguistic content is semantically expressed by tokened sentences at that context. Assessors infer that the speaker is conversationally implicating a particular metalinguistic proposition by engaging in the pragmatic reasoning process described in Sect. 3. Second, my account predicts whether a certain context is likely to involve metalinguistic negotiation. An assessor may engage in the reasoning process that leads her to reconstruct a metalinguistic proposition whenever the speaker would otherwise violate a Gricean maxim. The sorts of contexts where a speaker would violate a Gricean maxim include those where there is already agreement about the relevant properties of the items in question, those where a well-informed speaker would not believe the non-metalinguistic proposition expressed by her utterance, and those where the assessor is unable to identify any proposition expressed by the speaker’s utterance. Third, my account predicts which metalinguistic views will be conveyed at a particular context. The assessor reconstructs a metalinguistic proposition concerning the aptness of using some (normally, focused) expressions tokened by the speaker to convey a contextually determined proposition about the items denoted by other (normally, unfocused) expressions.

Additional appealing features of the account are that, firstly, it holds that metalinguistic propositions are conveyed during metalinguistic disputes. This feature is integral to an account’s potential to handle complex metalinguistic disputes, such as the ones that might emerge for my examples (3a)–(3e). Secondly, it draws on an independently developed analysis of expression focus. This feature is appealing due to the compelling connections with expression focus, and because it provides motivation to accept the account independent of considerations related to metalinguistic negotiation. As discussed in Sect. 1, Plunkett and Sundell (2019) express doubts about the potential to situate an account of metalinguistic negotiation within a Gricean framework. The account detailed in the current paper is therefore far from a trivial consequence of Gricean pragmatics.

## Footnotes

1 Note that this intuitive characterisation is not the definition of ‘verbal dispute’ that Chalmers arrives at. Different criteria for a dispute’s being verbal have been proposed by Hirsch (2005), Sider (2006), Sidelle (2007), Balcerak Jackson (2014), Jenkins (2014), Belleri (2018), Abreu Zavaleta (2020). For current purposes, an intuitive characterisation is sufficient.

2 In the current paper, I take an *expression* to be either an output of a grammar or an inscription or utterance that an individual is using to realise an output of the grammar. Following Potts (2007), an *output of a grammar* is a triple with the form: $\langle \Pi ; \Sigma ; \alpha : \sigma \rangle$, where $\Pi$ is a phonological representation, $\Sigma$ is a syntactic representation, and $\alpha$ is a semantic representation of type $\sigma$. Hence ‘died’ is an expression, as is the grammar output . When I describe an occurrence of an expression as *denoting* a certain item, I mean that the semantic representation in the relevant output of the grammar denotes that item.

3 Since the proposed analysis will predict that contexts with certain features are likely to involve metalinguistic negotiations, my neutrality centres on whether the contexts associated with particular disputes will have these features. As an anonymous reviewer helpfully points out, this neutrality allows me to avoid some contentious issues, such as whether a given dispute (say, one about whether composite objects exist) should be classified as a metalinguistic negotiation even when its participants reject this characterisation (see the discussion of ‘speaker error’ in Plunkett and Sundell 2019; Belleri 2020).

4 For instance, Ludlow (2014) argues that virtually all conversations involve negotiation about the use of language. While a non-‘mainstream’ linguistic theory might entail an adequate account of metalinguistic negotiation, it will predict that far more contexts involve metalinguistic negotiations than Plunkett and Sundell’s examples would suggest.

5 I invoke *certain purposes* in anticipation of the form that I attribute to the metalinguistic propositions that individuals convey (see Sect. 2.2).

6 I thank Matthew McKeever for raising this objection.

7 Some of the most significant work in the vast literature on focus includes: Chomsky 1971; Jackendoff 1972; Selkirk 1984; Rooth 1985; Kratzer 1991; Krifka 2007; Büring 2016. Note that it need not always be possible to identify exactly which items comprise the relevant alternatives, especially when an occurrence of a sentence is presented without a rich context. For instance, Bolinger (1961, p. 87) observes that ‘in *Let’s have a pícnic* [where ‘picnic’ is focused], coming as a suggestion out of the blue, there is no specific contrast with *dinner party*, but there is a contrast between picnicking and anything else the group might do’.

8 ‘Rutabaga’ and ‘neeps’ are respectively the American English and Scots words for the vegetable called ‘swede’ in England.

9 Note that the rules for marking focus do not always require every focused expression to be vocally emphasised. For instance, the focus of B’s utterance below may include ‘athlete’, despite the fact that the vocal emphasis falls on ‘not’: This is the case because expressions that are already *given* in the context—such as ‘athlete’ in a context in which A’s utterance has been issued—undergo *deaccenting* when they are included in a larger focus (Büring 2016, p. 28). Hence the second occurrence of ‘athlete’ in (i) may count as both employed in a metalinguistic usage and included in a focus, despite initial impressions otherwise. In contrast, if the clause-final vocal emphasis consists of ‘SecreTARiat’, then the rules for marking focus do not allow ‘athlete’ to be included in the focus of A or B’s utterances.

10 The standards that fix an aptness relation are typically influenced by factors like what is deemed polite, other discourse participants’ language-use, or speakers’ further aims (say, to obey the Gricean maxims, to use the minimum number of words, or to offend hearers). The flexibility of aptness relations makes them ideal for the analysis of metalinguistic negotiations. For, firstly, Sundell (2011, pp. 275–279) presents examples where participants in metalinguistic disputes disagree about the propriety of an expression due to its associated presuppositions, implicatures, degree of formality, contextual thresholds, and so on. This motivates an aptness relation that may hold or fail to hold between expressions and propositions due to syntactic, semantic, pragmatic, or even phonological properties of those expressions. Secondly, a uniform account of all metalinguistic disputes—both descriptive and normative—is desirable, due to parsimony considerations and because a metalinguistic dispute may vary over time in its status as a metalinguistic negotiation. This motivates an aptness relation that may hold between expressions and propositions due to factors related to how the expressions *are* used at the relevant circumstance, or those related to how the expressions *should be* used at that circumstance. For current purposes, I assume that when a speaker utters a sentence at a context *c* that expresses or implicates a metalinguistic proposition, it is *c* that determines the relevant standards and circumstance of evaluation for the aptness relation that contributes to that proposition.

11 The semantic type of the expressions employed in non-metalinguistic usages will determine whether their denotation is the function $\alpha$ or the argument $\beta$ in the embedded proposition.

12 At this point, the concern might arise that participants in many metalinguistic negotiations will communicate propositions for which the aptness relation is determined by distinct contextually relevant standards, thereby ‘talking past each other’ in a manner that undercuts the purported substantiveness of their dispute (I thank an anonymous reviewer for raising this point). First, the likelihood of such cases is lower than might be expected, since a speaker’s intentions need not be the primary determiner of the standards. For instance, if A intends to communicate that *e* is apt for conveying *p* because discourse participants *do* use *e* to convey *p*, and B intends to communicate that *e* fails to be apt for conveying *p* because discourse participants *should not* use *e* to convey *p* (say, because it is impolite), then this need not guarantee that each disputant will communicate a claim to which their intended standards contribute. Second, if some cases arise where speakers convey metalinguistic propositions for which distinct standards fix the aptness relation, then we may recover substantiveness by allowing the possibility of a ‘metametalinguistic negotiation’: disputants attempt to change how discourse participants use expressions, in order to cause the aptness relation determined by a later context to include the expression-proposition pairs that they envisage. On one hand, there might be motivation to endorse a non-propositional view of this negotiation (see Sect. 1), so as to avoid a potential regress of metalinguistic propositions involving higher-order aptness relations that apply to aptness relations. Crucially, the arguments that motivate the propositional view about metalinguistic negotiations conducted in the object language do not apply here: there are no overtly tokened sentential operators that appear to affect speakers’ views about appropriate aptness relations (see Sect. 2.1). On the other hand, as a different reviewer observes, a propositional analysis of ‘metametalinguistic negotiations’ might be justified on the grounds that cognitive limitations are likely to guarantee that no more than a few levels of higher-order aptness relations would ever be involved in communication.

13 Note that I do not commit myself to the view that assessors’ reasoning conforms to these discrete stages. One might wonder whose perspective determines if a dispute is metalinguistic: a hearer participating in the dispute or a dispute-external assessor. In cases where a participant in a dispute understands a speaker to conversationally implicate a metalinguistic proposition, then this is sufficient to render the dispute metalinguistic. Similarly, when an assessor reflects on a dispute lacking real participants and takes an imaginary speaker to implicate a metalinguistic proposition, then this suffices to render the envisaged occurrence of the dispute metalinguistic. More complex cases arise when a dispute-external assessor holds that a hearer participating in a real dispute has understood the speaker to implicate a metalinguistic proposition, but the hearer denies this charge. In such a scenario, it is the way in which the hearer actually understands the speaker that determines whether the dispute is metalinguistic. However, there could be cases where an external assessor has a better insight into the hearer’s actual pragmatic reasoning process than the hearer himself. For this reason, the current proposal takes no fixed position on whether a dispute should be classified as metalinguistic when an external assessor attributes a reasoning process to the hearer that the hearer rejects (see fn. 3).

14 This perspective receives empirical support; for instance, Tian and Breheny (2015) describe experimental data indicating that we infer contextual information ‘when we engage in conversation, *and* when we read speakerless out-of-context sentences ... Sentence comprehension can never be independent from context’ (p. 28).

15 This latter scenario might be thought to cohere poorly with a traditional Gricean approach, where an assessor must first identify the proposition expressed in order to access implicated content. However, many neo-Gricean frameworks (e.g., see Hirschberg 1985, p. 38) allow Gricean reasoning processes to be initiated by the mere utterance of a sentence. Also note that the potentially metalinguistic nature of the dispute is already reflected in step (i) of the reasoning process, because the assessor may be aware (and may assume that the speaker is aware) that their preferred meanings for an expression uttered by the speaker diverge; I am grateful to an anonymous reviewer for emphasising this point.

16 For empirical evidence that sentences are assigned some focus even when presented with no indication of the intended focus, see Fodor 2002, Frazier and Gibson 2015. For discussion of the idea that clause-final constitutents are the ‘unmarked’ choice of focus for English subject-verb-object sentences, see Lambrecht 1994, Carlson et al. 2009

17 Discourse participants’ inability to identify a unique contextually determined item that contributes to the information that they communicate is a common phenomenon (see the discussion of contextual domain restriction, gradable adjectives, demonstratives, etc., in Glanzberg 2007; King 2014). Plausibly, discourse participants are at least aware of a collection of suitably salient items, as indicated by their abilities to respond affirmatively or negatively when asked whether the speaker intended to communicate each of a variety of metalinguistic propositions.

18 Here is an example: It is natural for A to infer that B is communicating the metalinguistic proposition that ‘athlete’ (as opposed to the putatively mispronounced ‘afflete’) *is apt* for conveying that Secretariat *fails to have* the salient property (say, being a human proficient at sport).

19 For those who assess (2a)–(2f) relative to contexts where there is the chance of disagreement about some relevant properties of the items in question, there will be no pressure to reconsider the assumption that the speaker is conveying the non-metalinguistic proposition expressed by her utterance. An example of such a context would be one where both participants in (2b) agree about the criteria that an individual must meet in order to count as an athlete—say, outperforming at least $50\%$ of rivals that one has competed against—but disagree over whether Secretariat meets these criteria.

20 There are two reasons for this inference. First, assessors are likely to understand the focus of (2b) to consist of ‘athlete’ due to its clause-final position (see fn. 16), and Sect. 2.2 argued that focus generally indicates the expression that is employed in a metalinguistic usage. Second, since Plunkett and Sundell (2013) describe the metalinguistic construals that they are interested in before presenting any of their examples, they prime assessors to attend to this construal.

21 The operators and constructions involved in (3a)–(3e) mean that assessors are more likely to doubt that the speaker is proposing to add to the common ground a non-metalinguistic proposition than assessors are when (2a)–(2f) are presented out of context. The reason is that it is difficult to imagine ordinary contexts for such utterances where no maxims are violated. For the speaker of, say, (3e) would not only be proposing to add an uninformative proposition to the common ground (relative to a context where participants are mutually aware of the relevant properties of tomatoes), but would be proposing to add a proposition that a well-informed speaker would be unlikely to believe (i.e., that tomatoes vary over time with respect to their possession of certain individual-level properties). Hence the speaker risks violating the first maxim of Quality, even for contexts where there is sufficient doubt about the relevant properties to prevent the violation of the first maxim of Quantity.
